# Inflammatory Measures in Depressed Patients With and Without a History of Adverse Childhood Experiences

**DOI:** 10.3389/fpsyt.2018.00610

**Published:** 2018-11-27

**Authors:** Karin de Punder, Sonja Entringer, Christine Heim, Christian E. Deuter, Christian Otte, Katja Wingenfeld, Linn K. Kuehl

**Affiliations:** ^1^Charité – Universitätsmedizin Berlin, Corporate Member of Freie Universität Berlin, Humboldt Universität zu Berlin, and Berlin Institute of Health (BIH), Institute of Medical Psychology, Berlin, Germany; ^2^Department of Pediatrics, University of California, Irvine, Irvine, CA, United States; ^3^Development, Health and Disease Research Program, University of California, Irvine, Irvine, CA, United States; ^4^Department of Biobehavioral Health, College of Health and Human Development, Pennsylvania State University, University Park, PA, United States; ^5^Charité – Universitätsmedizin Berlin, Corporate Member of Freie Universität Berlin, Humboldt Universität zu Berlin, and Berlin Institute of Health (BIH), Department of Psychiatry and Psychotherapy, Berlin, Germany

**Keywords:** acute-phase protein, childhood adversity, childhood maltreatment, depression, inflammation, pro-inflammatory cytokine

## Abstract

**Background:** Major depressive disorder (MDD) is a complex psychiatric condition with different subtypes and etiologies. Exposure to adverse childhood experiences (ACE) is an important risk factor for the development of MDD later in life. Evidence suggests that pro-inflammatory processes may convey this risk as both MDD and ACE have been related to increased levels of inflammation. In the present study, we aimed to disentangle the effects of MDD and ACE on inflammation levels.

**Methods:** Markers of inflammation (plasma interleukin(IL)-6 and high sensitive C-reactive protein (hsCRP) concentrations, white blood cell (WBC) count and a composite inflammation score (CIS) combining all three) were assessed in 23 MDD patients with ACE, 23 MDD patients without ACE, 21 healthy participants with ACE, and 21 healthy participants without ACE (mean age: 35 ± 11 (SD) years). None of the patients and participants was taking psychotropic medication. ACE was assessed with the Early Trauma Inventory (ETI) and was defined as moderate to severe exposure to sexual or physical abuse.

**Results:** Group differences in the different inflammatory measures were observed. MDD patients with ACE showed significantly higher IL-6 concentrations (*p* = 0.018), higher WBC counts (*p* = 0.003) and increased general inflammation levels as indicated by the CIS (*p* = 0.003) compared to healthy controls. In contrast, MDD patients without ACE displayed similar inflammation levels to the control group (*p* = 0.93).

**Conclusion:** We observed elevated inflammation in MDD patients with a history of ACE, which could indicate a subtype of “inflammatory depression”. Accordingly, MDD patients with ACE might potentially benefit from anti-inflammatory therapies.

## Introduction

Major depressive disorder (MDD) is a frequent and heterogenic disorder and despite numerous studies performed over the last decades, there are still inconsistencies in clinical findings regarding the pathological mechanisms contributing to the development of MDD (1). Therefore, greater understanding of the biological processes and pathways underlying the pathophysiology of depression is of key importance for the development of early interventions and personalized therapies.

Adverse childhood experiences (ACE) have been shown to predispose to the development of MDD later in life (2, 3) and in addition induce greater risk for acquiring several somatic conditions, including cardiovascular disease (CVD) (3). A large body of evidence suggests that ACE is linked to a chronic pro-inflammatory state in adulthood (4–6). Alterations in the dynamics of the neuroendocrine stress response likely contribute to the manifestation of a pro-inflammatory immune phenotype in these individuals (7). It has been suggested that stressors occurring early in life can be biologically embedded through epigenetic modifications in stress-related genes (8) and program the immune system to become hyper-responsive in response to challenge with diminished sensitivity to the inhibitory effect of glucocorticoids (9).

Chronic inflammation is characterized by elevated levels of pro-inflammatory cytokines, acute phase proteins, and increases in white blood cell (WBC) numbers (10–12). Several studies reported higher circulating levels of inflammatory mediators, such as the pro-inflammatory cytokine IL-6 and the acute phase-protein C-reactive protein (CRP) (4, 5), and increased WBC counts (13, 14) in adults exposed to early adversity. Because chronic inflammation is associated with both ACE and several physical and psychiatric conditions, including MDD (15–18), it has been proposed as a key mechanism through which severe stress exposure during childhood can influence health outcomes throughout the lifespan (6, 19). This notion is further supported by the observation that increased activation of pro-inflammatory pathways (reflected by increased circulating levels of CRP and IL-6) precedes the development of depressive symptoms (17), and by studies that report that inflammation is more pronounced in a subgroup of MDD patients that are exposed to ACE (20–23).

The overall goal of the present study was to replicate previous findings regarding associations between ACE, MDD and inflammation, and to further disentangle the effects of MDD and ACE on inflammation using a well-controlled, full factorial design including four carefully diagnosed groups of healthy participants and MDD patients with and without a history of ACE. None of the patients and participants was taking psychotropic medication. We hypothesized accumulative effects of MDD and ACE on inflammation levels.

## Material and methods

### Participants

Patients and healthy participants were recruited by public postings and from our specialized affective disorder unit at the Department of Psychiatry and Psychotherapy, Campus Benjamin Franklin, Charité -Universitätsmedizin Berlin. All participants provided written informed consent. Healthy participants and outpatients received monetary compensation for their participation. The study was approved by the local ethical committee.

Depressed patients were included if they fulfilled criteria for MDD as assessed with the Structured Clinical Interview for DSM-IV axis I (SCID-I) (24) to validate psychiatric diagnoses. In addition, current depressive symptoms were captured by the Montgomery Asberg Depression Rating Scale (MADRS) (25, 26) and the Beck Depression Inventory (BDI) (27).

ACE was assessed by using a semi-structured interview, the Early Trauma Inventory (ETI) (28, 29), and was defined as repeated physical or sexual abuse at least once a month over one year or more before the age of 18.

In the MDD groups, schizophrenia, schizoaffective disorder, bipolar disorder, depressive disorder with psychotic features, dementia, eating disorders, panic disorder, alcohol or drug dependence led to exclusion. Healthy participants with and without ACE were free of any current mental disorder. Further exclusion criteria for all participants were CNS relevant diseases, neurological diseases, severe somatic diseases, diabetes type 1 and 2, steroid diseases, hypertonia, current infections, pregnancy and the intake of psychotropic medication. Physical health criteria were checked by physical examination, clinical interview and a complete blood count (CBC).

The study sample comprised 23 MDD patients with ACE (MDD+/ACE+), 23 MDD patients without ACE (MDD+/ACE–), 21 participants with ACE but no current, or lifetime MDD (MDD–/ACE+) and 21 participants with no current or lifetime MDD and no childhood adversity (MDD–/ACE–, healthy comparison group).

### Study protocol

All patients and participants underwent one study visit including psychiatric and medical diagnostic by physical examination, blood sampling and clinical interviews including SCID-I and MADRS as well as assessment of ACE using the ETI. Afterwards they completed a MDD related questionnaire (BDI). Blood samples were sent immediately to the laboratory of the Institute of Medical Psychology, Campus Mitte, Charité–Universitätsmedizin Berlin, Germany, and to the Labor Berlin–Charité Vivantes GmbH, Berlin, Germany, for further analyses.

### Inflammatory measures

Plasma IL-6 concentrations were analyzed using a commercially available high sensitivity ELISA kit (eBioscience), according to the manufacturer's instructions. The limit of detection was 0.007 pg/ml. The intra- and inter-assay coefficients of variability for plasma IL-6 measurements were 10 and 12%, respectively. Plasma hsCRP concentrations were analyzed using a commercially available Instant ELISA kit (eBioscience), according to the manufacturer's instructions. The limit of detection was 3 pg/ml. The intra- and inter-assay coefficients of variability for plasma hsCRP measurements were 6 and 8%, respectively. WBC counts were obtained from a standard clinical complete blood count panel using a Sysmex XN 1000 (Sysmex).

### Statistics

General linear models and Chi^2^ tests were used to compare groups concerning demographics and clinical data (see Table 1). *Post hoc* tests (Bonferroni) were conducted when applicable. IL-6 and hsCRP measures were first log transformed to normalize distributions. Since IL-6 and hsCRP concentrations and WBC count are established measures of pro-inflammatory activity, represent three biologically related components of inflammation [i.e., (a) pro-inflammatory cytokines (b) acute phase proteins and (c) increased WBC numbers], and inter-correlated with each other (all r's >0.2), we combined these measures into a single composite measure. Principal-component analysis identified one single factor, the composite inflammation score (CIS), accounting for 48% of the variance in analyte determinations. A common factor takes full advantage of the predictive values of the three measures, while minimizing measurement errors of the single components (21).

**Table 1:** Demographic and clinical characteristics of healthy participants and depressed patients without ACE (MDD–/ACE–, MDD+/ACE–) and healthy participants and depressed patients with ACE (MDD–/ACE+, MDD+/ACE+).

	**MDD–/ACE– (*n* = 21)**	**MDD+/ACE– (*n* = 23)**	**MDD–/ACE+ (*n* = 21)**	**MDD+/ACE+ (*n* = 23)**	**Statistics (GLM, Chi^2^)**
Age (SD)	33.90 (9.77)	32.61 (11.74)	34.05 (10.53)	38.09 (11.36)	*p =* 0.36
Sex (m/f)	8/13	5/18	6/15	9/14	*p =* 0.55
BMI (SD)	23.23 (3.56)^a, b^	21.49 (2.87)^a^	23.79 (3.14)^a, b^	25.05 (2.93)^b^	*p =* 0.003
Smoking (y/n)	4/17 ^a^	5/18 ^a^	7/14 ^a, b^	14/9 ^b^	*p =* 0.012
Educational level (%)					
Lower/Intermediate	23.8%	21.7%	28.6%	47.8%	*p* = 0.21
Upper Secondary School	76.2%	78.3%	71.4%	52.2%
**DEPRESSIVE SYMPTOMS**					
BDI (SD)	1.05 (1.56)^a^	25.41 (8.96)^b^	4.64 (4.75)^a^	26.81 (8.62)^b^	*p* < 0.001
MADRS score (SD)	0.62 (0.81)^a^	28.26 (5.71)^b^	1.67 (1.83) ^a^	27.41 (8.01)^b^	*p* < 0.001
**ADVERSE CHILDHOOD EXPERIENCES**					
ETI sum score (SD)	12.67 (21.13) ^a^	195.39 (205.48)^a^	619.62 (371.74)^b^	752.09 (482.57)^b^	*p* < 0.001

a, b *Groups with values that do not share a superscript within the same line of text are significantly different from each other. ACE, Adverse childhood experiences; BMI, Body mass index; BDI, Becks Depression Index; ETI, Early Trauma Interview; GLM, General linear model; MADRS, Montgomery Asberg Depression Rating Scale; MDD, Major depressive disorder; SD, Standard deviation*.

General linear models were used to compare groups regarding inflammatory measures. In order to investigate the groups effects on inflammatory measures in more detail, and because we expected the lowest inflammation levels in the control group, we studied a priori defined contrasts between the controls and the three study groups.

Many potential cofounders were excluded by design (see above). However, additional adjusted analysis included covariates that differed significantly between the four groups (i.e., BMI and smoking see Table 1).

Data analysis was performed using the SPSS statistical software (SPSS 23.0, Inc., Chicago, IL, USA). The significance level was set at *p* < 0.05 for all applied analysis.

### Missing data

Complete blood counts were missing for 8 individuals (4 MDD+/ACE+, 2 MDD+/ACE–, 1 MDD–/ACE+ and 1 MDD–/ACE–). For IL-6, a measurement was missing for 1 MDD patient without ACE (MDD+/ACE–). General linear models indicated there were no group differences regarding the number of missing biological measurements (*p* = 0.42).

## Results

### Sample characteristics

Table 1 summarizes group demographics and clinical characteristics. In accordance with our recruitment, MDD patients and healthy participant with ACE (MDD+/ACE+, MDD–/ACE+) had significantly higher total ETI scores compared to the MDD patients without ACE (MDD+/ACE–) and the control group (MDD–/ACE–). MDD patients with and without ACE did not differ in depression severity and both groups had higher depression scores compared to healthy individuals with and without ACE. MDD patients with ACE had a higher BMI compared to MDD patients without ACE and smoked more than healthy controls. No group differences were observed in age, sex and educational level.

### Inflammatory measures

**Figure 1:**
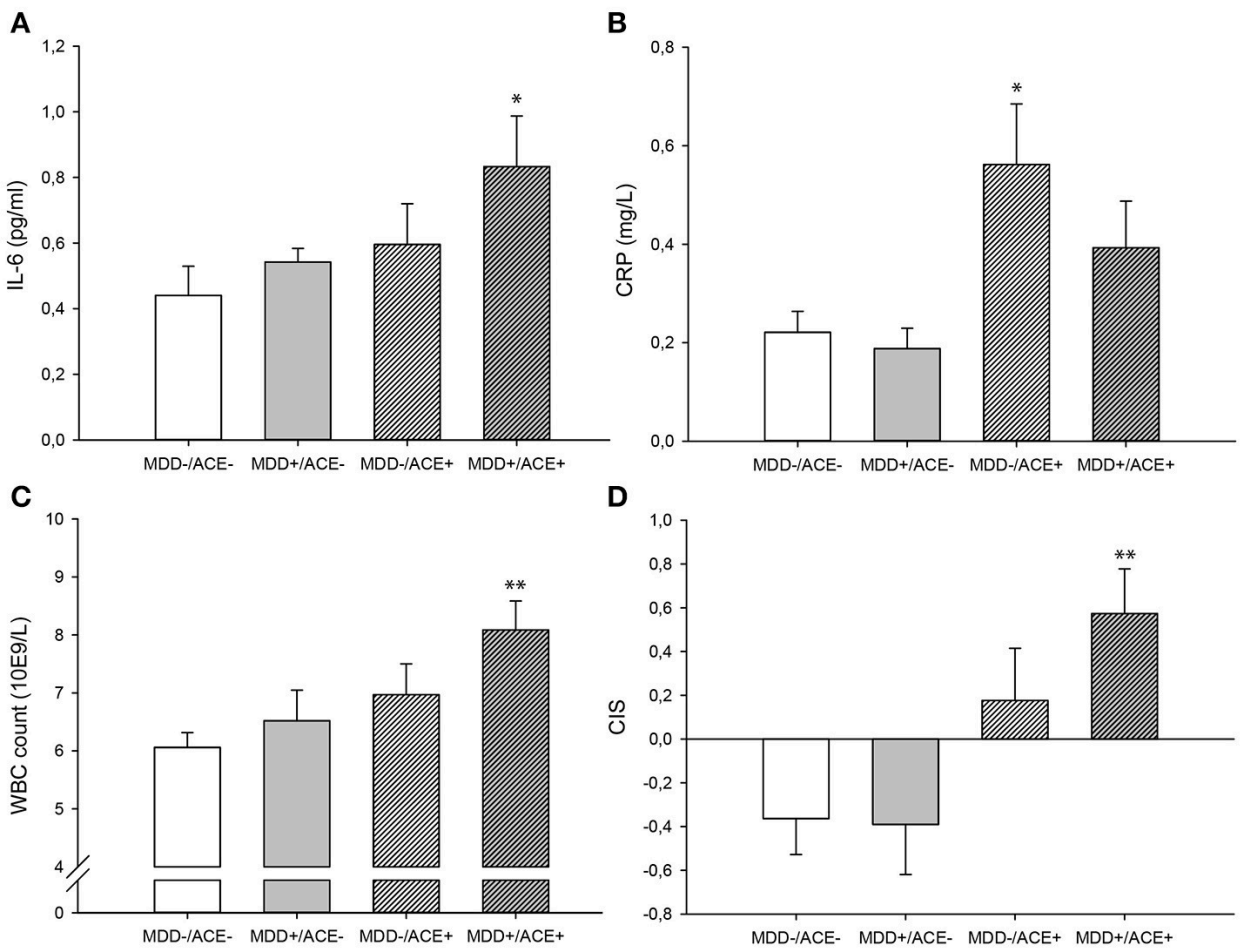
Untransformed and unadjusted mean group values for **(A)** Il-6, **(B)** hsCRP, **(C)** WBC count and **(D)** the unadjusted mean group CIS (±SE). *p-value < 0.05, **p-value < 0.01 in comparison to the control group (MDD–/ACE–). ACE, Adverse childhood experiences; CIS, Composite inflammation score; MDD, Major depressive disorder; WBC, White blood cell.

As presented in Table 2, we identified significant group effects on IL-6 [*F*_(3, 83)_ = 3.32, *p* = 0.024, η^2^ = 0.11], CRP concentration [*F*_(3, 84)_ = 3.10, *p* = 0.031, η^2^ = 0.10] and WBC count [*F*_(3, 76)_ = 3.44, *p* = 0.021, η^2^ = 0.12]. To investigate these effects in more detail, we studied a priori defined contrasts between controls and the different study groups (see Table 3). We observed significantly higher IL-6 concentrations (*p* = 0.018) and WBC counts (*p* = 0.003) in MDD patients with ACE compared to healthy controls, also after controlling for BMI and smoking (IL-6, *p* = 0.044; WBC count, *p* = 0.048). CRP levels were significantly higher in healthy individuals with ACE (*p* = 0.031). However, this effect was no longer significant after controlling for BMI and smoking (*p* = 0.052). Untransformed and unadjusted mean group values for IL-6, hsCRP and WBC counts are presented in Figures 1A–C.

**Table 2:** Mean values of the inflammatory measures of healthy participants and depressed patients without ACE (MDD–/ACE–, MDD+/ACE–) and healthy participants and depressed patients with ACE (MDD–/ACE+, MDD+/ACE+).

	**MDD–/ACE–**	**MDD+/ACE–**	**MDD–/ACE+**	**MDD+/ACE+**	**GLM**
IL-6 (Ln) pg/ml (SE)	−1.28 (0.25)	−1.44 (0.30)	−1.12 (0.29)	−0.43 (0.14)	*F*_(3, 83)_ = 3.32 *p* = 0.024
hsCRP (Ln) mg/L (SE)	−1.96 (0.22)	−2.12 (0.21)	−1.20 (0.29)	−1.49 (0.23)	*F*_(3, 84)_ = 3.10 *p* = 0.031
WBC count 10^9^/L (SE)	6.01 (0.27)	6.52 (0.53)	6.97 (0.53)	8.09 (0.50)	*F*_(3, 76)_ = 3.44 *p* = 0.021
CIS (SE)	−0.36 (0.16)	−0.39 (0.23)	0.18 (0.24)	0.57 (0.20)	*F*_(3, 75)_ = 4.76 *p* = 0.004

*ACE, Adverse childhood experiences; Composite inflammation score; GLM, General linear model, MDD, Major depressive disorder; SE, Standard error; WBC, White blood cell*.

**Table 3:** Mean differences in inflammatory measures between each of the three study groups (MDD+/ACE–, MDD–/ACE+ and MDD+/ACE+) and control group (MDD–/ACE–) before (unadjusted) and after adjusting for BMI and smoking.

	**MDD–/ACE–**	**MDD+/ACE–**	**MDD–/ACE+**	**MDD+/ACE+**
**IL-6 (Ln) pg/ml**				
*Unadjusted (95% CI)*	–	−0.16 (−0.87, 0.54) *p* = 0.65	0.16 (−0.55, 0.87) *p* = 0.65	0.85 (0.15, 1.54)^*^ p = 0.018
*BMI & Smoking (95% CI)*	–	−0.10 (−0.83, 0.63) *p* = 0.79	0.14 (−0.58, 0.86) *p* = 0.70	0.77 (0.02,1.53)^*^ p = 0.044
**hsCRP (Ln) mg/L**				
*Unadjusted (95% CI)*	–	−0.16 (−0.83, 0.51) *p* = 0.64	0.76 (0.07, 1.45)^*^ p = 0.031	0.47 (−0.20, 1.14) *p* = 0.17
*BMI & Smoking (95% CI)*	–	−0.04 (−0.72, 0.63) *p* = 0.90	0.68 (−0.01, 1.36) *p* = 0.052	0.21 (−0.50, 0.92) *p* = 0.56
**WBC count 10**^9^**/L**				
*Unadjusted (95% CI)*	–	0.51 (−0.80, 1.82) *p* = 0.44	0.96 (−0.36, 2.28) *p* = 0.15	2.08 (0.74, 3.42)^**^ p = 0.003
*BMI & Smoking (95% CI)*	–	0.60 (−0.65, 1.84) *p* = 0.34	0.69 (−055, 1.93) *p* = 0.27	1.33 (0.01, 2.65)^*^ p = 0.048
**CIS**				
*Unadjusted (95% CI)*	–	−0.27 (−0.62, 0.56) *p* = 0.93	0.54 (−0.05, 1.13) *p* = 0.073	0.94 (0.34, 1.54)^**^ p = 0.003
*BMI & Smoking (95% CI)*	–	0.11 (−0.48, 0.69) *p* = 0.72	0.47 (−0.10, 1.04) *p* = 0.11	0.66 (0.05, 1.26)^*^ p = 0.034

*ACE, Adverse childhood experiences; CI, Confidence interval; CIS, Composite inflammation score; MDD, Major depressive disorder; WBC, White blood cell*.

* indicates significant difference compared to the control group, p-value < 0.05;

** *p-value < 0.01*.

There was a significant group effect on the CIS [*F*_(3, 75)_ = 4.76, *p* = 0.004, η^2^ = 0.16, Table 2]. Contrasts between the controls and the different study groups showed that inflammation levels tended to be increased in healthy participants with ACE (*p* = 0.073, Table 3) and were significantly higher in MDD patients with ACE compared to healthy controls (*p* = 0.003, Table 3), also after controlling for BMI and smoking (*p* = 0.034, Table 3). Unadjusted mean group CIS values are depicted in Figure 1D.

## Discussion

With the present study we aimed to disentangle the effects of MDD and ACE on alterations in levels of inflammation by using well-controlled, defined and discrete groups of adults with and without a history of ACE and an MDD diagnosis. Confirming our hypothesis, we observed the highest inflammation levels in MDD patients with a history of ACE. These results replicate prior research showing that inflammation is elevated in a subgroup of MDD patients exposed to ACE (21). Our results are also in line with previous studies reporting that elevations in inflammatory measures observed in MDD patients are associated with childhood trauma (22, 23, 30). Another study comparing cytokine levels between healthy controls and MDD patients with and without a history of ACE found the highest levels of 13 different cytokines in the ACE exposed MDD patients (31). However, in contrast to our findings, no increases in plasma levels of IL-6 were observed in this subgroup of MDD patients. A possible explanation for this discrepancy could be the use of different assay methodology.

Group differences were not completely homogenous regarding the different inflammatory measures that we assessed. While IL-6 concentrations and WBC counts were elevated in MDD patients with ACE, higher CRP concentrations were seen in healthy individuals exposed to ACE compared to healthy controls. This last finding is supported by a recent meta-analysis suggesting that the association between childhood trauma and inflammatory measures, including CRP, is not moderated by the presence of a psychiatric diagnosis (5). However, our finding that CRP is increased in healthy individuals exposed to ACE was no longer significant after adjusting for BMI and smoking. As previously reported by others, unhealthy lifestyle factors like increased BMI and smoking are associated with ACE (32) and have been shown to have an effect on inflammation (33, 34). Therefore, these lifestyle factors could have contributed to the observed elevations in CRP concentrations.

Altogether, the data presented here support the hypothesis that ACE might be a risk factor for developing a MDD later in life, and that this risk is partly mediated by increases in activation of pro-inflammatory pathways (19). In line with this, inflammation levels did not differ between MDD patients without ACE and healthy controls, suggesting that biological mechanisms, other than inflammation, might play a more prominent role in the pathogenesis of depression in these patients. Although previous research has shown increased inflammatory measures in depression (15, 17, 18), in most studies the effects of ACE have not been taken into account.

ACE is not only a risk factor for the development of depression. Also other psychiatric disorders, such as post-traumatic stress-disorder (PTSD), anxiety disorders, and bipolar disorder have been associated with a history of ACE (35, 36) and are as well related to increased inflammation (37, 38). However, until now, only few studies systematically investigated the separate and interactive effects of disease status and a history of ACE on inflammation (39–41). Therefore, future research should attempt to further identify the role of ACE in activating inflammatory pathways in these psychiatric conditions.

Findings from this study are limited by the use of a cross sectional design and the relatively modest sample size, which might have led to insufficient power. Also our study sample included relatively more female than male participants, indicating that our results might be impacted by gender bias. However, the groups did not differ significantly regarding the female to male ratio. In addition, the number of immune parameters that we assessed was limited, and future studies should include additional measures of acute-phase proteins, cytokines, and immune cell characteristics in order to gain better understanding of the biological processes and pathways underlying the inflammation-related pathophysiology of depression in patients with and without a history of ACE.

Our study has several strengths. The study sample consisted of four carefully diagnosed groups which allowed us to disentangle the effects of MDD and ACE on inflammation. Furthermore, none of the patients and participants was taking psychotropic medications. All patients and participants received detailed diagnostics and a physical examination. Our findings of increased levels of inflammation in MDD patients (and to a lesser extent in healthy individuals) exposed to ACE, in this rather young study sample further emphasize the clinical importance of our results, since elevated inflammation is a risk factor implied in the development of somatic disorders like CVD (3, 42). Moreover, the pro-inflammatory state observed in depression also has consequences for treatment success, since patients with elevated inflammation are less likely to respond to conventional antidepressants (43, 44).

In summary, in this well-controlled study, we replicated findings from prior research suggesting accumulative effects of MDD and ACE on a more pro-inflammatory state, while inflammation levels did not differ between MDD patients without ACE and healthy controls. These findings suggest that a subgroup of MDD patients with a history of ACE might benefit from an anti-inflammatory intervention.

## Ethics statement

This study was carried out in accordance with the recommendations of the Charité's Ethics Committee with written informed consent from all subjects. All subjects gave written informed consent in accordance with the Declaration of Helsinki. The protocol was approved by the Charité's Ethics Committee.

## Author contributions

KdP carried out the laboratory assays, participated in the data analyses, and drafted the manuscript. SE designed the study participated in the analysis and interpretation of study findings and provided editorial assistance. CH participated in the data analysis and provided editorial assistance. CD participated in the conduction of the study, interpretation of study findings and provided editorial assistance. CO participated in the study design, analysis and interpretation of study findings and provided editorial assistance. KW participated in the study design, analysis and interpretation of study findings and provided editorial assistance. LK conceived of and designed the study, conducted the study, participated in the analysis and interpretation of study findings, drafted portions of the manuscript and provided final editorial oversight.

### Conflict of interest statement

The authors declare that the research was conducted in the absence of any commercial or financial relationships that could be construed as a potential conflict of interest.
